# Exploring psychological counselors' perspectives on the characteristics and dynamics of online counseling

**DOI:** 10.3389/fpsyg.2025.1708309

**Published:** 2026-01-12

**Authors:** Ahmet Buğa, Mehmet Özdemir, Fatih Bozbayındır

**Affiliations:** 1 Department of Educational Sciences, Gaziantep University, Gaziantep, Türkiye; 2 Graduate School of Educational Sciences, Gaziantep University, Gaziantep, Türkiye

**Keywords:** online psychological counseling, therapeutic relationship, client self-disclosure, counselor professional development, ethical considerations

## Abstract

**Introduction:**

This study examines online psychological counseling with a focus on the therapeutic relationship and clients' self-disclosure behaviors, as perceived by experienced psychological counselors. With the increasing use of digital platforms in mental health services, understanding the opportunities and limitations of online counseling has become increasingly important.

**Methods:**

A qualitative phenomenological research design was employed. The study sample consisted of 14 psychological counselors who had experience conducting online counseling sessions. Data were collected through semi-structured interviews and analyzed using content analysis with the support of MAXQDA software.

**Results and conclusion:**

The findings indicate that online psychological counseling provides key benefits, especially greater accessibility and temporal-spatial flexibility. Nonetheless, technical difficulties (e.g., internet disruptions) and ethical issues related to confidentiality and trust remain important challenges. The strength of the therapeutic alliance was shaped by clients' personal characteristics and their adaptation to digital platforms. Online settings were perceived to facilitate self-disclosure among shy, privacy-sensitive clients or those who struggle with face-to-face interactions, while limited nonverbal cues were seen as restricting emotional communication and potentially weakening the therapeutic relationship. Self-disclosure was closely linked to perceived safety, relationship quality, and access to private, distraction-free environments. Participants also associated preferences for online counseling with demographic and personal factors such as age, socioeconomic status, and anxiety. In addition, online counseling supported counselors' professional development by enabling work with diverse clients, strengthening cultural competence, and improving time management. Overall, while online psychological counseling offers substantial opportunities, its effectiveness depends on individualized approaches, strong technological infrastructure, and clear ethical guidelines.

## 1 Introduction

According to the American Counseling Association (ACA), psychological counseling refers to the application of mental health and developmental principles through cognitive, affective, behavioral, and interactive intervention strategies to promote individuals' wellbeing, personal and professional growth, and to address psychological disorders (Hackney and Cormier, 2008). Within this helping process, numerous psychological theories have been developed to understand human nature and psychological difficulties, including psychodynamic, Adlerian, behavioral, existential, person-centered, cognitive-behavioral, reality, Gestalt, feminist, solution-focused, and narrative approaches (Corey, 2005). All of these therapeutic frameworks share the common goal of fostering individuals' development and addressing the challenges they encounter. One of the critical considerations in the helping process concerns how psychological assistance is delivered to the individual. Traditionally conducted in a face-to-face setting, counseling processes—like many other professional practices—have been compelled by the rapid advancement of technology to make critical decisions regarding the integration of technological tools into psychological counseling services (Abney and Maddux, 2004). Various digital platforms and media have been utilized, either individually or in combination, to deliver psychological support, including SMS-based interventions (Bauer et al., 2003), mobile phones, virtual reality applications (Goss and Anthony, 2009), email and chat rooms (Maples and Han, 2008), video conferencing systems (Castelnuovo et al., 2003), structured CD-ROM programs (Stallard et al., 2011; Proudfoot et al., 2003; Khanna and Kendall, 2010), and web-based interactive programs (Buga and Hamamci, 2016; O'Kearney et al., 2006; Spence et al., 2011). Among these innovations, online therapy or online counseling has long been recognized as a method for providing psychological assistance. Online counseling refers to a process in which a professional therapist interacts with a client via the internet to address psychological concerns, offering guidance and emotional support (Dinçyürek and Uygarer, 2012). This form of counseling, which is considered the closest equivalent to traditional face-to-face therapy, has nonetheless required significant adaptations in the nature and dynamics of the therapeutic relationship. In traditional face-to-face counseling, the therapeutic relationship is strengthened by non-verbal cues and physical proximity; however, their absence in online psychological counseling significantly alters the relational dynamics (Zeren, 2017). Online psychological counseling refers to counseling services conducted between geographically distant individuals (Bloom, 2008) while maintaining the professional boundaries of the client–counselor relationship (Mallen and Vogel, 2005). In other words, it is a method of providing psychological assistance to individuals or groups through technological communication channels (Barak and Grohol, 2011; Richards and Viganò, 2012). Online counseling is often preferred in situations such as financial constraints, time limitations, geographical barriers, illness, physical disabilities, childbirth, or a lack of access to appropriate facilities (Poyrazli and Can, 2020; Turkish Psychological Counseling and Guidance Association, 2011). The COVID-19 pandemic has further underscored the importance of online psychological counseling, as counselors increasingly turned to digital platforms to reach clients who were unable to access services in person (Poyrazli and Can, 2020). Compared to face-to-face counseling, online psychological counseling operates under a distinct set of dynamics. Factors such as the reliance on technological tools, the absence of physical co-presence, concerns regarding privacy and ethics, and the nature of client–therapist interactions all play a critical role in shaping the therapeutic relationship in online settings (Berger, 2016; Simpson and Reid, 2014). These differences highlight the need for research examining how the therapeutic relationship is established and maintained in online counseling contexts.

According to the American Psychological Association (2023), self-disclosure in the therapeutic process refers to the act of sharing personal information about oneself with others. The counseling process, grounded in confidentiality and trust, provides a context in which clients disclose their experiences, explore problems in depth, and work toward solutions. Within this process, client self-disclosure functions as a facilitating factor that enhances therapeutic progress (Çetinkaya Yildiz, 2019). Self-disclosure, defined as the extent to which an individual reveals private and personal information, has also been conceptualized as a dimension of personality (West and Zingle, 1969). A simple example of self-disclosure is when an individual introduces themselves by stating their name and subsequently shares personal details. Through this process, individuals communicate their unique thoughts and experiences to others (Dai et al., 2015). Recent technological advancements have enabled individuals to engage in self-disclosure in online settings, making this an increasingly important communication skill to be cultivated both face-to-face and online (Bucholtz, 2013). However, clients' self-disclosure processes in online environments may differ from those observed in traditional counseling. For instance, a study by Ünal-Karagüven and Kamak (2023) conducted with adolescents revealed that although adolescents preferred to disclose more in face-to-face counseling, self-disclosure was still possible online when certain conditions were met. The study revealed that participants perceived interpersonal trust, shyness, and the need for social connection as factors shaping how adolescents make sense of and engage in self-disclosure.

Client self-disclosure is a decisive factor in sustaining the therapeutic process, and fostering this behavior largely depends on the therapist's empathetic understanding and reassuring attitude. However, the client's willingness and ability to disclose are shaped by a range of factors, including individual differences, cultural norms, and life experiences (Denizli, 2009). Although self-disclosure lies at the heart of the therapeutic relationship, its healthy progression requires mutual effort and understanding from both the client and the therapist. The ability to disclose is therefore crucial for an effective counseling process; conversely, difficulties in self-disclosure can hinder progress and complicate the therapeutic work (Ünal-Karagüven and Kamak, 2023).

The therapeutic relationship is one of the most fundamental elements of the psychological counseling process, referring to the trust-based, supportive, and collaborative bond established between the counselor and the client (Norcross and Wampold, 2011). In online counseling, skills such as content reflection, feeling reflection, summarizing, and maintaining a focus on the here and now are considered essential for fostering this relationship (Haberstroh et al., 2008). Moreover, in the context of online psychological counseling, factors such as the absence of non-verbal cues, reliance on written communication, issues of anonymity, confidentiality, and the establishment of trust emerge as critical variables influencing the quality of the therapeutic relationship (Gülden and Polat, 2022).

The therapeutic relationship differs from other types of interpersonal interactions in that it is goal-oriented and time-limited, functioning as a problem-solving process that concludes after a defined period (Kottler and Brown, 2000). While it shares many qualities with other effective human relationships, the therapeutic relationship also possesses unique characteristics. Similar to face-to-face counseling, the absence of a therapeutic relationship in online psychological counseling is likely to reduce the effectiveness of the intervention for most clients (Mallen et al., 2005). Nevertheless, studies by Cook and Doyle (2002) and Prado and Meyer (2006) indicate that a strong therapeutic bond can be established even in online text-based or email counseling. Research has demonstrated that a strong therapeutic relationship significantly impacts both the effectiveness of the psychological counseling process and the client's motivation for change (Horvath et al., 2011). In the context of online psychological counseling, despite the constraints imposed by the physical environment, the quality of the therapeutic relationship remains a critical determinant of counseling effectiveness (Sucala et al., 2012). Simpson and Reid (2014) further emphasized that while a strong therapeutic relationship can indeed be cultivated online, achieving this requires therapists to adapt their technological competencies and communication strategies accordingly.

A systematic review by Sucala et al. (2012) found that the therapeutic relationship in online psychological counseling can be as effective as that in face-to-face counseling. The review highlighted that clients generally perceive e-therapy as supportive and satisfactory, and that the quality of the therapeutic relationship is positively associated with treatment outcomes. However, a subsequent study by the same research group (Sucala et al., 2013) reported that therapists often lack confidence in establishing relationships in online counseling and face challenges related to non-verbal communication skills, such as conveying empathy. Similarly, Kotera et al. (2021) identified both positive and challenging experiences among therapists providing online therapy during the COVID-19 pandemic. Technological limitations, the absence of a dedicated therapeutic space, and insufficient professional training are among the factors that make establishing a therapeutic relationship in online environments particularly challenging. A comparative study by (Mercadal Rotger and Cabré 2022) found that face-to-face therapy is significantly more effective than online therapy in terms of building a therapeutic relationship. In the Turkish context, Pulat's (2022) qualitative thesis reflected how both clients and counselors made sense of the online counseling process across multiple dimensions, including the establishment of the therapeutic relationship, the management of ethical boundaries, challenges in achieving emotional depth, and the role of technical infrastructure, while also presenting practice-oriented insights. Collectively, these studies underscore the importance of structured support, targeted professional training, and a technology-sensitive approach to ensure the sustainability of the therapeutic relationship in online psychological counseling.

The growth and widespread adoption of technology and internet usage have facilitated the emergence and expansion of online psychological counseling services, extending beyond traditional face-to-face counseling contexts (Korkmaz and Sen, 2018). With the continuous advancement of these technological capabilities, online counseling has attracted increasing attention. Despite this rapid growth and widespread interest, the literature on online psychological counseling services remains limited (Piri, 2011). While research in Türkiye is relatively scarce, the topic has been examined internationally for many years (Barnett, 2005; Cohen and Kerr, 1998; Cook and Doyle, 2002; Mallen and Vogel, 2005; Murphy et al., 2009). Key areas of study include the quality and effectiveness of the therapeutic relationship in online psychological counseling, as well as clients' experiences of self-disclosure in online vs. face-to-face contexts (Zeren, 2017; Ünal-Karagüven and Kamak, 2023).

In this context, examining the experiences of psychological counselors who provide or have experience in online counseling—specifically how they establish the therapeutic relationship, observe clients' self-disclosure processes, and interpret body language, gestures, and facial expressions within the therapeutic bond—has become an important research focus. Additionally, understanding how these processes are shaped by the individual characteristics of clients in online counseling is critical. This study aims to identify the fundamental elements involved in structuring the therapeutic relationship and building trust in online psychological counseling, as well as to explore clients' behaviors during their self-disclosure processes. Accordingly, the research seeks to answer the following questions:

1. What are psychological counselors' views regarding the general characteristics of the online psychological counseling process?
2. What are psychological counselors' views on therapeutic alliance, self-disclosure, body language, client characteristics, and professional development within the context of the online psychological counseling process?

## 2 Method

### 2.1 Research model

The present study aimed to explore the nature of online counseling through the perspectives of counselors with experience in this modality, focusing specifically on their experiences regarding the therapeutic relationship and clients' self-disclosure during counseling sessions. To achieve this aim, a qualitative research approach was employed (Seggie and Bayyurt, 2017), as it allows for an in-depth examination of emerging problems and solutions within the context of a rapidly changing and evolving field. Within the phenomenological approach, this research design provides an opportunity to explore in depth how individuals interpret, articulate, and make sense of their lived experiences (Patton, 2018), as well as what they perceive through those experiences (Gedik, 2016). Among the various phenomenological designs, descriptive phenomenology was employed in this study. This design emphasizes the identification and articulation of lived experiences to enhance understanding of particular entities, objects, or phenomena (Kiral, 2021). The descriptive phenomenological design was deemed appropriate for this study because the primary aim was to examine in detail the experiences of participating psychological counselors regarding online counseling, with a specific focus on their perceptions of the counselor-client relationship and the degree to which clients felt comfortable disclosing personal issues.

### 2.2 Participants

The study group consists of 14 psychological counselors, including eight women and six men, who work in various institutions such as middle schools, high schools, special education, and rehabilitation centers, and who work individually without being affiliated with any institution. The participant group of the study was formed using snowball sampling, one of the purposive sampling methods. Snowball sampling is an important type of sampling in terms of reaching individuals who can be a source of data related to the problem created by the researcher (Yildirim and Simsek, 2021). First, several people who could provide data for the research were interviewed. Subsequently, these individuals were asked: who would you recommend we talk to about this topic? Who could provide us with more information on this subject? These individuals then referred the researchers to other individuals who could provide data. In this way, the researchers attempted to reach individuals who were more appropriate for the research topic in terms of the reliability of the research. In this study, data saturation was considered to occur at the point where themes began to recur and the emergence of new information diminished, as is typical in qualitative research. This approach is grounded in the experimental work of Guest et al. (2006). Consistent with this understanding, the interview data in the present study were analyzed thematically, and it was observed that no new themes emerged after the final interviews. Therefore, it was concluded that data saturation had been reached. When presenting the findings, psychological counselors were given code names such as Veysel, Nazan, and Sadiye, which do not correspond to their real names. The demographic information of the psychological counselors who participated in the study is presented in Table 1.

**Table 1:** Demographic characteristics of the psychological counselors participating in the study.

**Code name**	**Gender**	**Age**	**Professional seniority**	**Education level**	**Number of individuals receiving in-person counseling**	**Number of individuals receiving online counseling**	**Counseling groups**	**Region of individuals receiving online counseling**	**Theoretical background**	**Institution of employment**
Veysel	Male	33	9 Years	MA	325	15	Adolescent	Different	CBT	Secondary school
Murat	Male	28	4 Years	MA	150	20	Child-adolescent	Same-different	ACT	Independent counselor
Sedat	Male	27	2 Years	MA	70	18	Adolescent-adult	Same-abroad	Eclectic	Independent counselor
Nazan	Female	26	3 Years	PHD	15	10	Adolescent-adult	Same-different	CBT	Independent counselor
Lale	Female	32	7 Years	BA	130	10	Child-adolescent	Same	CBT	Independent counselor
Betül	Female	26	2 Years	BA	100	15	Adult	Same	Gestalt	High school
Mehmet	Male	28	4 Years	BA	200	25	Adolescent	Different	CBT	High school
Seda	Female	31	6 Years	BA	250	30	Adolescent-adult	Same-different	CBT	Independent counselor
Ekrem	Male	28	3 Years	BA	110	7	Adolescent-adult	Same	Eclectic	Secondary school
Zeliha	Female	27	5 Years	MA	65	12	Adolescent	Same	CBT	Special education and rehabilitation center
Tülay	Female	29	3 Years	BA	100	17	Adolescent	Same-different	CBT	Secondary school
Harun	Male	28	4 Years	MA	170	6	Adolescent-adult	Same-different	CBT	Secondary school
Hilal	Female	26	2 Years	BA	30	5	Adolescent	Different	Eclectic	Municipality
Sadiye	Female	25	2 Years	BA	125	14	Adolescent-adult	Same-different	CBT	High school

CBT, Cognitive behavioral therapy; ACT, Acceptance and commitment therapy.

Table 1 indicates that the study group consisted of 14 psychological counselors who had experience in both face-to-face and online counseling. All participants held degrees in Psychological Counseling and Guidance (PCG); eight had completed an undergraduate degree, five held a master's degree, and one had a doctoral degree. The participants' ages ranged from 25 to 33, and their professional experience varied between 2 and 9 years. The group included eight females and six males, indicating a balanced gender distribution. The participants were employed in various institutional settings, including schools (primary, secondary, and high school), municipalities, and private special education centers. Most of the counselors worked predominantly with adolescents and adults, while some also provided services to children. The number of clients receiving online counseling from the participants ranged from 5 to 30. In terms of theoretical background, Cognitive Behavioral Therapy (CBT) was the most commonly adopted approach, followed by eclectic, Gestalt, and Acceptance and Commitment Therapy (ACT) approaches. Additionally, the clients receiving counseling services resided in the same city, in different cities, or abroad, indicating that participants' online counseling experiences extended across diverse geographical and cultural contexts.

Despite working in different institutional settings, the participants shared several professional characteristics that allowed the data obtained from the interviews to be compared and interpreted coherently. All participants held degrees in Psychological Counseling and Guidance (PCG) and were actively engaged in providing psychological counseling services. Each participant had experience in both face-to-face and online counseling practices and had regularly implemented online counseling in their professional roles. A majority of the participants delivered these services within educational institutions or private counseling centers. These shared professional features enabled the data gathered from diverse work environments to be evaluated within a common analytical framework.

### 2.3 Data collection instrument

In line with the aim of the study, a semi-structured interview form was employed to collect data. After identifying the relevant contexts and phenomena of the research, a nine-item semi-structured interview form was developed. The literature on the topic was reviewed, and the form was refined based on feedback from three experts. Initially, the form contained eight questions; subsequently, an additional question was added to obtain more in-depth information, and some existing questions were revised. Several questions were modified to ensure clarity and to facilitate the collection of more detailed data. A pilot interview was conducted to test the comprehensibility of the questions. The interview questions were designed to explore how participants perceived and made sense of the identified phenomena within the context of online psychological counseling. For example, one question asked whether conducting counseling online affects clients' self-disclosure behaviors and, if so, how. Other questions similarly aimed to elicit observations and experiences related to the therapeutic relationship and clients' self-disclosure in the context of online counseling.

### 2.4 Credibility, transferability, dependability, and confirmability studies

In this study, credibility was enhanced by frequently incorporating participants' direct quotations. According to Shenton (2004), the inclusion of participants' verbatim statements allows readers to evaluate the extent to which the identified themes are grounded in the data. To further strengthen credibility, participants were provided with a comfortable environment in which they could express themselves freely, and they were informed about the purpose and scope of the research prior to the interviews. Participant validation, expert review, and prolonged engagement with participants are regarded as essential strategies for enhancing credibility in qualitative research (Başkale, 2016). In this context, in-depth insights were obtained from participants through interviews totaling 400 min, conducted with psychological counselors during the data collection process. After the coding process was completed, member checking was performed to verify the findings and enhance the study's credibility (Lincoln and Guba, 1985; Onwuegbuzie and Leech, 2007). Furthermore, to ensure the credibility of the study, the opinions of two subject matter experts were sought at each stage of the research process, as expert consultation is known to strengthen the credibility of qualitative studies (Lincoln and Guba, 1985). During the research process, the steps of data collection, participant selection, and data analysis must be documented in detail to ensure the consistency of the study (Yildirim and Simsek, 2021). Accordingly, these processes were carefully monitored and systematically recorded in the present study. To ensure transferability, researchers are expected to provide sufficient contextual information about the fieldwork (Shenton, 2004). Therefore, comprehensive information regarding the participants and the research process was included in this study.

### 2.5 Data collection and analysis

In qualitative research, the primary aim is not to generalize findings to a larger population but to provide a detailed exploration of specific phenomena (Creswell, 2016). Accordingly, semi-structured interviews were conducted with a total of 14 psychological counselors who formed the study group. Prior to the interviews, participants were informed about the study and appointments were scheduled. Data were collected through face-to-face interviews as well as online platforms, including Zoom and Google Meet. Environments were selected to ensure that both the researcher and participants could communicate comfortably. Participants were assured that the collected data would be used solely for scientific purposes, thereby providing a secure and confidential setting. All interviews were recorded using either written notes or audio recording devices. The interviews lasted, on average, between 25 and 30 min.

In this study, the three-stage coding process (open coding, axial coding, and selective coding) developed by Strauss and Corbin (1990) was followed in the data analysis. In this context, during the open coding phase, the data were analytically broken down, and events and interactions were classified with conceptual labels to form categories. Subsequently, in the axial coding stage, these categories were associated with their subcategories. Finally, in the selective coding phase, all categories were integrated around a “core category,” thereby defining the main phenomenon of the study and ensuring theoretical coherence. Throughout these processes, theme development was achieved by interpreting the concepts emerging from coding at a higher level of abstraction. In addition, to ensure researcher reflexivity, constant comparison with the data was conducted to ensure that the interpretations were grounded in the participants' experiences.

This study adopts descriptive phenomenology, grounded in the Husserlian tradition, as its philosophical orientation. Descriptive phenomenology seeks to understand individuals' lived experiences as they are, without interpretation, while remaining faithful to their essential nature and uncovering the common structural meanings that underlie these experiences (Giorgi, 2012). Within phenomenological research, content analysis facilitates the systematic identification and presentation of themes that reflect participants' experiences, thereby contributing to a comprehensive yet descriptive understanding of the phenomenon. Content analysis is widely recognized as an appropriate analytical strategy in qualitative descriptive studies (Sandelowski, 2000; Bengtsson, 2016). In this regard, it allows data to remain at a surface level of meaning and to be described without altering the essence of participants' experiences. Accordingly, in the present study, content analysis was applied in alignment with the epistemological orientation of descriptive phenomenology; the process of organizing data into meaningful thematic units enabled the identification of the essence of participants' online counseling experiences. In this process, audio recordings were first converted into raw data in a digital format. Subsequently, the responses to each interview question were analyzed using MAXQDA, a qualitative data analysis software, and common codes and themes were identified. During the coding and theme development process, relevant concepts from the literature were taken into consideration. Similar codes and themes were merged to create new, more comprehensive codes and themes. The resulting codes and themes were designed to align closely with the core focus of the study, ensuring that they were meaningful, coherent, and internally consistent.

## 3 Findings

**Figure 1:**
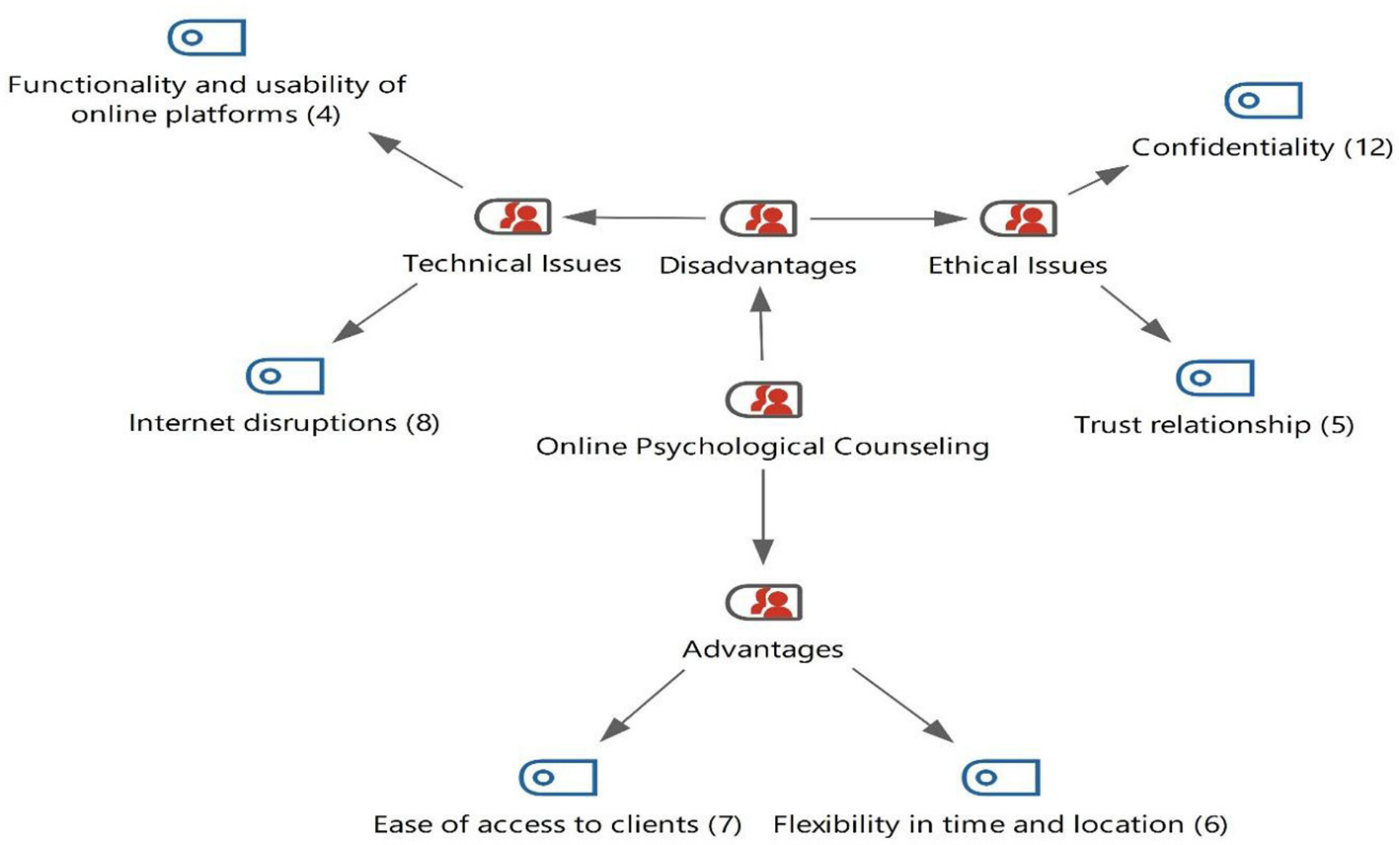
Perspectives of psychological counselors on online counseling.

This section presents the findings derived from the content analysis concerning the general characteristics of the online psychological counseling process, as summarized in Figure 1.

Figure 1 shows that teachers' views indicated that the contributions of online psychological counseling applications to stakeholders are expressed as ease of access for clients (*f* = 7) and flexibility in terms of time and place (*f* = 6). On the other hand, teachers also mentioned some disadvantages of online psychological counseling. These disadvantages emerged as confidentiality (*f* = 12), internet interruptions (*f* = 8), trust relationship (*f* = 5), and the functionality and usability of online platforms (*f* = 4).

When Figure 1 is examined, it becomes clear that participants' views on online psychological counseling emerged under two main categories: advantages and disadvantages. In explaining the advantages, counselors emphasized flexibility in terms of time and place, as well as the ease of reaching clients. Specifically, seven participants noted that they could easily reach clients, while six participants highlighted time and place flexibility as an important advantage.

On the other hand, the disadvantages were grouped into two subcategories: ethical issues and technical problems. Regarding technical problems, eight participants mentioned internet disconnections as a major challenge, whereas four participants reported difficulties related to the use of online platforms. In terms of ethical issues, twelve participants stated that confidentiality was the most significant concern in the counseling setting, five participants referred to challenges in building trust, and one participant noted the potential risk of self-harming behavior by clients.

According to participants' views, the most frequently mentioned disadvantage under technical problems was internet disconnection, while confidentiality was the most frequently reported issue under ethical concerns. Conversely, the most frequently emphasized advantage of online counseling was the ease of client accessibility. Below are some excerpts from participants regarding the technical problems associated with the disadvantages of online counseling.

For instance, one participant emphasized the potential impact of the platform used during online sessions:

Murat stated,

“*The platform used during online counseling can itself have an effect. For example, when conducting sessions via Zoom, the visible time limit may prevent the client from introducing new topics. In face-to-face sessions, there is no such time constraint; clients may not pay much attention to the clock and can bring up different issues. Toward the end of the session, unless the counselor intervenes, the client can continue to express themselves comfortably. On Zoom, however, even the time display at the top of the screen can influence the client.”*

This perspective underscores the potential impact of digital platforms on the counseling process. The constant visibility of time can induce subtle pressure on clients, limiting spontaneous disclosure and potentially affecting session depth.

Similarly, another participant highlighted the role of technical disruptions in shaping the counseling process: Zeliha shared,

“*The client's resources during counseling are very important. For instance, a sudden internet disconnection may distract the client, prevent them from remaining present in the moment, or even damage the counselor–client relationship.”*

This statement draws attention to how technological limitations can interrupt therapeutic flow, reduce emotional engagement, and compromise the continuity of the counselor–client alliance.

Regarding the disadvantages of online counseling, participants also expressed concerns about ethical issues. Selected excerpts are provided below:

Harun stated,

“*…there are inevitably some disadvantages as well. Especially during the initial stage of acquaintance and disclosure, problems may arise in building trust. From the client's perspective, it is difficult to trust someone they have never met in person and to share their secrets with them.”*

This highlights that establishing a therapeutic alliance may be more challenging in online settings, as the absence of physical presence can hinder the development of trust and openness—key components of effective counseling.

Similarly, Hilal shared,

“*To be honest, I used to conduct sessions at home in the evenings. I would wear headphones so that others in the house would not hear what the counselor said and to avoid disturbing them. Of course, I was in my own room, but while expressing my feelings, I was somewhat cautious, wondering whether someone else in the house might be listening. Having experienced this process myself as a client, I believe clients may also feel the same way.”*

This statement points to privacy concerns in online counseling, where the client's physical environment may compromise confidentiality and inhibit emotional expression.

Regarding the ease of reaching clients, some participants shared the following views:

Ekrem stated,

“*I have a client from abroad, for instance, from Germany. Online counseling removes such boundaries, which is a very significant advantage. Likewise, I also have clients from different cities—for example, from Ankara. These are serious benefits, as it becomes much easier to reach clients.”*

This statement underscores that online counseling eliminates geographical barriers, allowing counselors to work with clients regardless of their physical location, thereby increasing access to psychological services.

Similarly, Sadiye noted,

“*When working with test anxiety, online counseling is also very useful for us. The child may be living in another city, or at times we may not be able to reach a student otherwise. In such cases, online counseling makes access much easier.”*

This view highlights the practical advantage of online counseling in cases where in-person sessions would be difficult or impossible, thus supporting continuity of psychological intervention.

Within the theme of flexibility in time and space, participants' views are presented below:

Veysel stated,

“*In the online psychological counseling process, we save both distance and time. Otherwise, since the person is in a remote city, it is not really possible to meet face-to-face.”*

This statement emphasizes that online counseling offers an alternative solution for clients living in remote areas, eliminates geographical barriers, and makes psychological support more accessible.

Seda expressed,

“*To give a few examples, my military client cannot remain tied to a single location for face-to-face therapy due to constant reassignments. Similarly, a student who attends school spends very late hours there, so he has no time left for therapy. By receiving therapy at home, he saves the time he would otherwise spend on the road.”*

This view emphasizes that online counseling is particularly beneficial for clients with time constraints or mobile lifestyles, offering them greater convenience and continuity of care.

**Figure 2:**
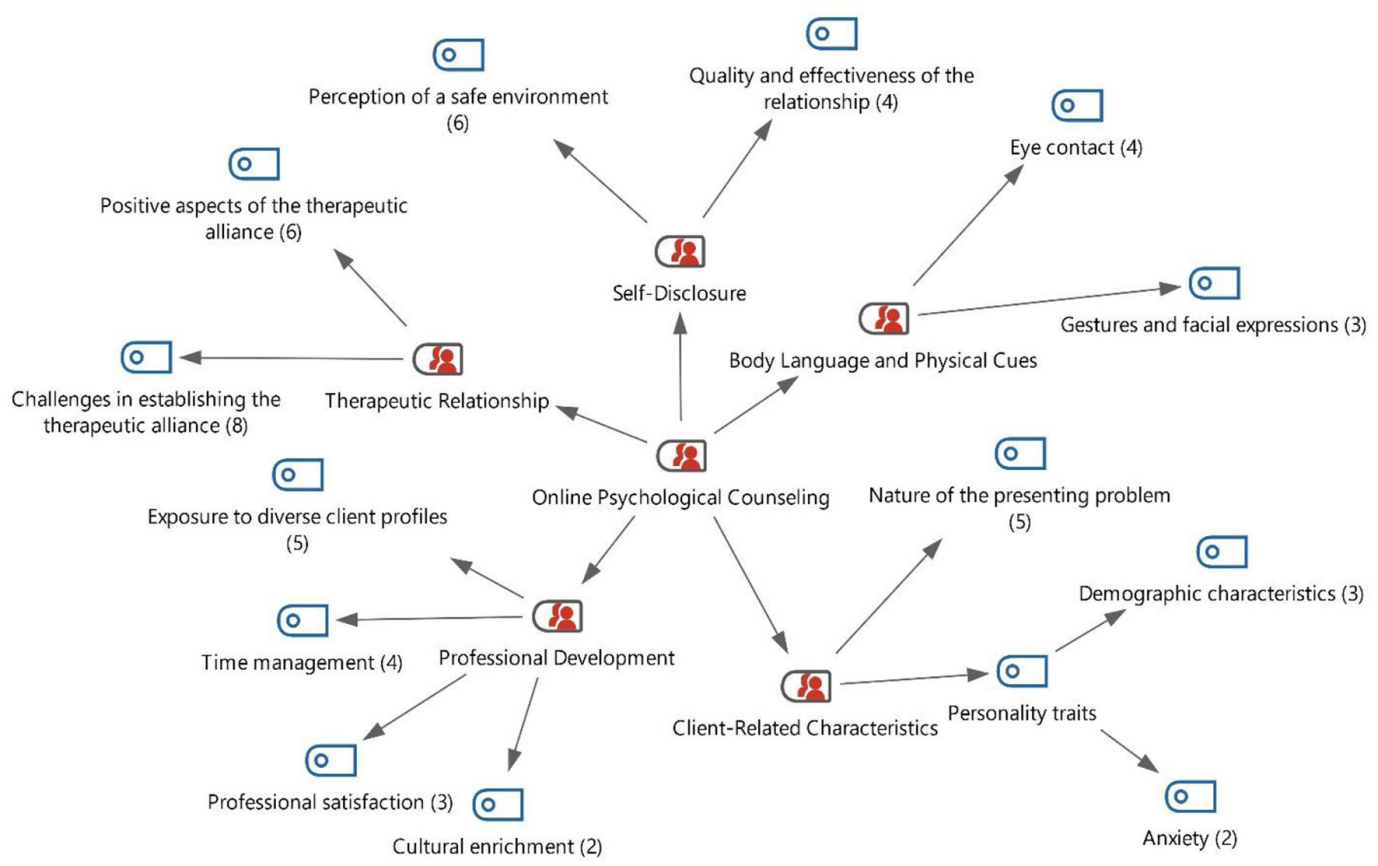
Findings on therapeutic relationship, self-disclosure, body language, client characteristics, and professional development in online psychological counseling.

In the second sub-problem of the study, participants were asked questions concerning the nature of the therapeutic relationship in online counseling. Based on participants' views, the findings related to the therapeutic relationship in online psychological counseling are presented in Figure 2.

Upon examining Figure 2, the findings related to online psychological counseling practices based on teachers' perspectives were grouped under five main themes: therapeutic relationship, body language and physical cues, self-disclosure, professional development, and client characteristics. Under the theme of therapeutic relationship, two categories emerged: challenges in establishing a therapeutic alliance (*f* = 8) and positive aspects of the therapeutic alliance (*f* = 6). Within the body language and physical cues theme, the codes of eye contact (*f* = 4) and gestures and facial expressions (*f* = 3) were identified. The self-disclosure theme included the perception of a safe environment (*f* = 6) and quality and effectiveness of the relationship (*f* = 4). Under professional development, findings were obtained related to working with exposure to client profiles (*f* = 5), time management (*f* = 4), professional satisfaction (*f* = 3), and cultural enrichment (*f* = 2). Finally, within the client characteristics theme, the codes associated with the nature of the presenting problem (*f* = 5), demographic characteristics (*f* = 3), and anxiety (*f* = 2) emerged.

Figure 2 shows that, based on participants' views, the therapeutic relationship in online counseling was examined under two categories: the positive aspects of the therapeutic bond in the online setting and the challenges experienced in establishing the therapeutic bond online. Of the 14 participants, six referred to the positive aspects of the therapeutic bond in online counseling, whereas eight participants emphasized the challenges encountered in establishing such a bond. In the category of challenges in forming a therapeutic bond online, highlighted by eight participants, it was noted that the absence of face-to-face interaction was understood to shape clients' perceptions and behaviors, with some participants making sense of this as clients acting as if they were in an artificial environment. Furthermore, participants indicated that because online counseling has not yet become fully widespread, clients may have difficulty expressing themselves comfortably on these platforms, which can hinder the establishment of a clear counselor–client relationship. Conversely, six participants stated that the bond established in online counseling could be healthier, particularly for individuals who have difficulty expressing themselves in face-to-face counseling, those with shy personalities, and those who fear being overheard or exposed when speaking to a stranger in an external environment.

Selected quotations from participants regarding the challenges of the therapeutic relationship are presented below.

Veysel stated,

“*Some clients have difficulty expressing themselves comfortably even in face-to-face sessions or do not take the process seriously, which can lead to significant problems in establishing a bond.”*

This statement highlights that certain clients' limited engagement or discomfort can hinder the formation of a strong therapeutic relationship, a challenge that may be amplified in online counseling settings.

Murat stated,

“*Because sincere communication cannot be fully established, I believe the therapeutic relationship is, above all, a human connection. First, this human relationship must be established before moving on to the therapeutic process. Due to the presence of a screen and the mediated nature of the interaction, the process of establishing this relationship takes longer compared to face-to-face counseling.”*

This statement highlights Murat's view that the mediated and less intimate nature of online platforms can delay the establishment of a therapeutic alliance compared to in-person sessions.

Hilal stated,

“*At least in terms of the therapeutic relationship, there used to be certain cues. For instance, when the doorbell rang and I welcomed the client—or the way they greeted me—it gave me subtle messages about their week. How did they arrive that day? What was their emotional state? Were they willing to attend the session? Were they anxious? Were they in a balanced mood? These observations offered me valuable insights. When I brought them into the room, I would give them 10–15 s to acclimate to the environment, to reconnect with me, and to take a breath. I believe that this was an advantage of face-to-face sessions, which cannot be fully replicated online.”*

This statement highlights that face-to-face counseling enables therapists to observe nonverbal cues and create a natural transition period that supports emotional readiness, an element that is largely absent in online counseling.

Regarding the positive aspects of the therapeutic bond established in online counseling, participants shared the following views:

Betül stated,

“*In the online setting, we conduct counseling in an environment where the client feels comfortable. In this context, we can establish the relationship in a more qualified manner.”*

This statement highlights that online counseling may foster a more effective therapeutic relationship by allowing clients to engage in a setting where they feel safe and at ease.

Sadiye stated,

“*In face-to-face counseling, we sometimes cannot establish the same level of relationship that we can observe more frequently in online counseling. In online counseling, the person is in their own safe environment, whereas in face-to-face counseling, they are within the boundaries of the counselor's space. In terms of the therapeutic relationship, feeling safe and opening up are significantly different between online and face-to-face counseling.”*

This statement highlights that clients' sense of safety and comfort in their own environment during online sessions may facilitate greater self-disclosure and strengthen the therapeutic bond compared to face-to-face settings.

Harun stated,

“*…this greatly reduces their concerns about whether someone else might see them. In such situations, it provides significant advantages in the therapeutic relationship.”*

This statement highlights that online counseling can reduce clients' privacy concerns, thereby supporting a stronger and more trusting therapeutic relationship.

Participants' views on self-disclosure behavior in the online psychological counseling process are presented in Figure 2. Upon examining Figure 2, it is observed that, based on the participants' perspectives, self-disclosure behavior in the online psychological counseling process is categorized into two main themes: a safe environment and the quality and efficiency of the relationship. Among these themes, the most frequently emphasized aspect was a safe environment, highlighted by six counselors. Four participants provided insights regarding the quality and efficiency of the relationship in the online setting. Selected quotations from participants regarding the sub-theme of a safe environment are presented below:

Murat stated,

“*During the online counseling process, if the client is situated within a family setting, I believe this can negatively affect self-disclosure. Compared to counseling conducted in a face-to-face office environment, this can be considered a disadvantage. However, if the client creates an appropriate environment for themselves during the online session, this disadvantage can be mitigated.”*

This statement underscores the critical role of a physically and psychologically safe setting in facilitating clients' willingness to disclose personal information during online counseling.

Lale added,

“*In online counseling, especially with certain adolescent groups, attention can easily be distracted. Audio issues may occur, and we sometimes need to repeat questions multiple times, which can hinder clients from opening up during the counseling process.”*

This comment highlights how technical and environmental distractions in online settings can impede clients' self-disclosure, emphasizing the need for optimal conditions to support effective counseling interactions.

Selected quotations from participants regarding the quality and efficiency of the relationship in self-disclosure are presented below:

Ekrem stated,

“*The quality and depth of the relationship established with the client can sometimes be lacking in online counseling, which may lead the client to avoid self-disclosure on many issues. Online psychological counseling can facilitate self-disclosure, particularly for clients exhibiting anxiety symptoms. In a safe environment, the client can express themselves more comfortably and easily; however, for some clients, the lack of face-to-face interaction may make it difficult to establish trust, and self-disclosure may not occur.”*

This statement emphasizes that while online counseling can facilitate self-disclosure for certain clients, particularly those with anxiety, the depth and quality of the therapeutic relationship are critical factors in determining whether clients feel sufficiently secure to open up.

Tülay added,

“*If a strong therapeutic relationship can be established, the client's self-disclosure behavior will increase positively.”*

This comment emphasizes the importance of establishing a strong and supportive counselor-client relationship in online environments to encourage self-disclosure.

Findings regarding the participants' body language and physical cues during the online psychological counseling process are given in Figure 2. When examining Figure 2, it is observed that the theme of body language and physical cues in online counseling is divided into two sub-themes: eye contact and gestures/facial expressions. Four participants emphasized the importance of eye contact, while three highlighted that gestures and facial expressions should be closely attended to during counseling sessions. The counselors stressed that, during online counseling, attention to body language and physical cues—particularly eye contact, gestures, and facial expressions—is essential. Four participants underscored the importance of monitoring whether the client maintains eye contact during therapy, while three participants emphasized that clients' body language and moment-to-moment emotional changes can significantly affect the counselor's understanding of them. Selected statements from participants regarding clients' gestures and facial expressions in online counseling are presented below.

Sedat stated,

“*In face-to-face counseling, we see the client directly. Even from the trembling of their foot or the way they move it, we can interpret that they are under stress or better understand which of our statements or theirs affected them. This can be a disadvantage in online counseling.”*

This statement highlights that the absence of direct physical observation in online counseling can limit the counselor's ability to accurately interpret subtle stress or emotional cues.

Harun added,

“*One of my adolescent clients used to shake their foot. At first, they thought it was normal, but as the sessions progressed, they realized that the foot shaking was due to anxiety. This awareness became a significant milestone in the therapy process. However, noticing this in an online counseling setting can be somewhat difficult.”*

This comment emphasizes that online counseling may make it challenging for counselors to detect subtle physical behaviors that are important for understanding clients' emotional states, which can impact the therapeutic process.

Selected quotations from participants regarding the importance of eye contact in online counseling are presented below:

Zeliha stated,

“*In the online environment, establishing mutual eye contact with the client, even through an artificial screen, is very important. You can understand their emotional state from their gaze or the way they avoid eye contact.”*

This statement highlights that eye contact remains a crucial non-verbal cue in online counseling, allowing counselors to assess clients' emotions despite the virtual setting.

Mehmet added,

“*I can distinguish my clients' emotional states based on whether or not they establish eye contact. I guide my interventions accordingly.”*

This comment emphasizes that monitoring eye contact enables counselors to adjust their approach and respond powerfully to clients' emotional needs during online sessions.

Some participants described how they made sense of clients' individual characteristics within the online psychological counseling process. The findings obtained from the content analysis are presented in Figure 2. As shown in Figure 2, the analysis revealed that clients' individual characteristics were primarily categorized into two sub-themes: general/personal traits and problem situations. Subsequently, the general/personal traits sub-theme was further divided into two codes: demographic factors and anxious client profiles. Five participants emphasized that clients' general and personal characteristics could significantly shape the course of online counseling. Among them, three specifically highlighted the importance of demographic factors (e.g., age, socioeconomic status). The remaining two participants noted that anxious clients often struggle to express themselves clearly, which can hinder the creation of an effective therapeutic environment. Furthermore, four participants underlined that the therapeutic alliance and the progress of sessions may vary depending on the client's specific problem situation. An illustrative statement provided by one of the participants regarding clients' problem situations is presented below.

Betül stated,

“*The intervention for a client with a severe problem differs from that for a client with a less severe issue. For example, an individual with a severe obsession may require more technical interventions, whereas routine daily interventions may be sufficient for someone experiencing exam-related stress.”*

This statement highlights that the intensity and type of intervention in online counseling should be tailored to the severity and nature of the client's concerns, emphasizing a flexible, client-centered approach.

Some participants' views regarding clients' general and personal characteristics are presented below.

Lale stated,

“*Especially when counseling an anxious individual, our work becomes more difficult. Because they constantly focus on past or future situations, it becomes almost impossible for the sessions to be beneficial for them.”*

This statement emphasizes that clients' personality traits, particularly high levels of anxiety, can significantly reduce the effectiveness of online counseling and make it difficult to achieve therapeutic outcomes.

Tülay stated,

“*The age of the clients also affects the online psychological counseling process. For instance, I would prefer face-to-face counseling for a younger child. However, with an adolescent, establishing a therapeutic bond online can be easier compared to the younger client.”*

This statement indicates that participants made sense of the suitability and effectiveness of online counseling in relation to demographic factors such as age, perceiving adolescents as more likely to engage comfortably in online settings.

Counselors were asked how online counseling contributed to their professional development. The findings obtained from participants' responses to this question are presented in Figure 2. Upon examining Figure 2, it is observed that, in response to the question regarding their professional development, three participants stated that they experienced growth in terms of professional satisfaction, while five participants emphasized the advantage of encountering diverse client profiles. In addition, two participants indicated that, alongside meeting different clients, they also had the opportunity to become acquainted with different cultures. Four consultants emphasized the importance of time management, noting that using specific online platforms for consulting sessions contributes to the development of time management skills. Based on the participants' views, the most frequently emphasized subtheme in relation to professional development through online counseling was exposure to diverse client profiles. Selected participant statements concerning the subthemes of diverse client profiles and cultural enrichment in online counseling are presented below.

Harun stated,

“*…when we conduct online counseling, we can even go beyond national borders. As I mentioned, I even had a client from Germany. Of course, working with clients from much farther away and from very different cultures and cities provides significant advantages.”*

This statement highlights that online counseling expands the reach of psychological services, allowing counselors to work with clients from diverse geographical and cultural backgrounds, thereby broadening their professional experience.

Murat stated,

“*…I believe it is advantageous in terms of expanding one's portfolio, working with clients facing different issues, and reaching people in various locations, which may also increase your visibility among their social circles.”*

This statement emphasizes that online counseling not only enhances professional development by providing exposure to diverse client cases but also helps increase the counselor's professional recognition across different communities.

Some participants' views on time management in the context of the online psychological counseling process are presented below.

Betül stated,

“*Online psychological counseling provides flexibility. Most importantly, in online counseling, the time is clearly defined—it starts and ends at a set hour. The boundaries are very clear because the session duration is fixed. Consequently, you learn to adjust according to the schedule, and your time management skills improve.”*

This statement emphasizes that online counseling inherently structures session times, which helps counselors develop better time management skills and ensures sessions are conducted efficiently.

Tülay stated,

“*I can adjust the sessions according to both myself and the client, which allows me to manage my time effectively. I can say it provides a significant advantage in using time economically.”*

This statement highlights that online counseling allows both the counselor and client to optimize scheduling, enhancing efficiency and making more productive use of time.

Some participants' statements regarding professional satisfaction in online psychological counseling are presented below.

Zeliha stated,

“*Having the opportunity to work with different groups positively affects my professional satisfaction.”*

This statement highlights that online counseling allows counselors to engage with a more diverse client base, which can enhance their sense of professional fulfillment.

Hilal stated,

“*I researched certain online platforms for psychological counseling and registered on them. Then, I connected with counselors there to learn how to reach clients and how to behave appropriately. This not only added to my professional experience but also created an additional career avenue for me. As I mentioned, I was always working face-to-face, and I realized I could continue counseling online, which opened a new door for me.”*

This statement emphasizes that online counseling provides professional growth opportunities and alternative career pathways, allowing counselors to expand their skills and reach beyond traditional face-to-face settings.

## 4 Discussion

The findings of this study revealed that online psychological counseling services should be evaluated from a multidimensional perspective, considering both their advantages and disadvantages. Participants' views on the advantages of online counseling were primarily centered on time and space flexibility and the ease of access to clients. This finding is consistent with previous studies highlighting the opportunities offered by online counseling in terms of accessibility. For example, Dilkes-Frayne et al. (2019) emphasized that online counseling provides 24/7 access and serves as an important resource, particularly for individuals seeking support outside regular working hours. Similarly, Moudatsou et al. (2024) noted that therapists viewed the spatial flexibility and cost-effectiveness of online counseling as favorable features. Supporting the results of the present study, other researchers (Amos et al., 2020; Ardi et al., 2017; Haryati, 2020; Kiriakaki et al., 2022; Mejah et al., 2019; Tuna and Haskan Avci, 2023; Yüksel Sahin, 2021; Zeren et al., 2022; Zhang and Liu, 2020; Yurayat and Tuklang, 2023) have similarly emphasized that online counseling provides temporal and spatial flexibility.

Technical problems were among the most frequently cited disadvantages in the findings of this study. The majority of participants considered internet connection interruptions to be a major barrier that disrupts the counseling process. This finding is consistent with the study conducted by (Eker and Genç 2025), which reported that internet outages create stress for clients, negatively affect the therapy process, and pose accessibility challenges for older adults. Likewise, other studies have identified connection problems and platform incompatibilities (Amos et al., 2020; Dilkes-Frayne et al., 2019; Donat Bacioglu and Onat Kocabiyik, 2019; Haryati, 2020; Moudatsou et al., 2024; Smith and Gillon, 2021; Tuna and Haskan Avci, 2023), lack of adequate hardware (Kiriakaki et al., 2022), insufficient technological infrastructure (Zhang and Liu, 2020), and software errors (Ardi et al., 2017) as key disadvantages of online counseling.

In the context of ethical issues, confidentiality emerged as the most prominent concern in this study. Confidentiality is one of the fundamental principles of online counseling (Ardi et al., 2017). The majority of participants stated that ensuring confidentiality in online counseling settings was the most challenging ethical issue. This finding aligns with the study of Chen et al. (2022), which reported that clients' willingness to seek online counseling decreases due to ethical concerns such as confidentiality and crisis intervention. Furthermore, Kiriakaki et al. (2022) highlighted that issues such as data security and identity verification create legal and ethical uncertainties in online counseling. Similarly, previous studies have emphasized confidentiality challenges in online counseling (Moudatsou et al., 2024; Tannous, 2017; Tuna and Haskan Avci, 2023), the sharing of documents in digital environments (Eker and Genç, 2025), and the lack of specialized counselor training as potential factors leading to ethical violations (Mejah et al., 2019). Conversely, some studies have suggested that online counseling can offer advantages in terms of confidentiality, particularly when addressing sensitive issues (Amos et al., 2020; Gülden and Polat, 2022; Mejah et al., 2019; Yurayat and Tuklang, 2023; Zhang and Liu, 2020). In this regard, confidentiality in online counseling may be perceived as either an advantage or a disadvantage, depending on the counselor's training and the security of the software or platform used. Overall, the findings of the present study largely converge with the existing literature on the advantages and disadvantages of online psychological counseling. The results indicate that, in order to expand the provision of online counseling services, both technological infrastructure should be strengthened and ethical standards should be clearly defined.

In this study, the quality of the therapeutic alliance in online psychological counseling was found to vary depending on clients' personal characteristics and their level of adaptation to the digital environment. Some participants reported that the therapeutic relationship established in an online setting provided a safer and more comfortable space for individuals who are shy, concerned about privacy, or who struggle with face-to-face communication. This finding is supported by Amos et al. (2020), who noted that online counseling offers a more comfortable environment for self-expression and enhances the sense of safety. Similarly, Smith and Gillon (2021) found that online counseling increases self-awareness and strengthens the therapeutic alliance. On the other hand, participants frequently highlighted the challenges of establishing a therapeutic bond online. In particular, the lack of face-to-face interaction may lead to the counseling setting being perceived as artificial and make it difficult for clients to express themselves. The limited availability of gestures, body language, and facial expressions during online counseling can hinder the counselor's ability to provide emotional feedback, thereby weakening the therapeutic relationship (Simpson and Reid, 2014). Zeren et al. (2022) and Haryati (2020) similarly emphasized that the absence of nonverbal communication in online settings can negatively affect the therapeutic alliance and limit clients' experience of intimacy. Moreover, Zeren (2017) reported that building a therapeutic relationship in online counseling requires greater attention and effort compared to face-to-face communication, particularly due to the lack of nonverbal cues such as body language, which may complicate the process of forming a strong therapeutic bond.

Clients' attitudes toward the online environment also emerged as an important factor influencing the quality of the therapeutic alliance. For instance, Alt et al. (2022) found that individuals who prefer to express their emotions in an online setting tend to have a more positive perception of online counseling. Similarly, Chen et al. (2022) identified clients' level of trust in the online environment as one of the most significant predictors of their willingness to engage in online counseling, noting that individuals with higher trust levels are better able to establish a therapeutic relationship. The reliability of counselors in online counseling sessions is therefore crucial for maintaining the quality of the process (Mulawarman et al., 2023; Yurayat and Tuklang, 2023). As Amos et al. (2020) pointed out, the lack of physical contact with the counselor may create a sense of distrust among clients. In this context, employing techniques that foster a sense of trust and facilitating clients' adaptation to the digital environment are critical for strengthening the therapeutic alliance. In conclusion, the quality of the therapeutic alliance in online psychological counseling varies according to clients' individual differences and their perceptions of the online environment. This finding highlights the need for online counseling services to be delivered in a more inclusive and flexible manner, as well as the importance of developing strategies to strengthen the therapeutic relationship in digital contexts.

The findings of this study revealed that clients' self-disclosure behavior in online psychological counseling is directly related to the safety of the counseling environment and the quality of the counselor–client relationship. Participants' emphasis on the presence of a safe setting indicates that privacy and psychological security are among the key determinants of clients' level of openness in online counseling. Similarly, previous studies (Eker and Genç, 2025; Tuna and Haskan Avci, 2023) reflected that participants made sense of the home environment as not always suitable for online counseling, as it was perceived to challenge privacy, create distractions, and limit open communication. In this regard, because the client's physical setting has a direct impact on the counseling process (Amos et al., 2020), creating a private and quiet environment is considered essential (Zhang and Liu, 2020). The quality and effectiveness of the relationship established in the online counseling environment also emerged as another crucial factor influencing clients' capacity for self-expression. Consequently, building a strong counselor–client relationship should be a priority in online counseling (Zhang and Liu, 2020). On the other hand, it should not be overlooked that online counseling may provide a safer and more comfortable space for expression for some clients. For instance, Amos et al. (2020) reported that online counseling services offer a more supportive and expressive environment, particularly for shy individuals and students experiencing social pressure. When these findings are evaluated together, it can be concluded that online counseling services can have different effects depending on the characteristics of the clients and that they must have a flexible structure to meet individual needs. Consequently, providing a safe environment and strengthening the quality of the counseling relationship are fundamental prerequisites for improving the functioning of online counseling services.

The findings of this study indicate that body language and physical cues are crucial components of therapeutic interaction in online psychological counseling. Participants' emphasis on eye contact, gestures, and facial expressions suggests that the limited availability of nonverbal communication in online settings may affect the counseling process. This observation aligns with the study by Zeren et al. (2022), which similarly highlighted that the lack of nonverbal communication in online counseling can weaken the therapeutic alliance and hinder the accurate understanding of clients' emotional states. Amos et al. (2020) also noted that nonverbal cues such as gestures and facial expressions are diminished in online counseling contexts.

Likewise, several studies have identified the lack of body language as a significant limitation in online counseling (Eker and Genç, 2025; Dilkes-Frayne et al., 2019; Haryati, 2020; Kiriakaki et al., 2022; Mejah et al., 2019; Moudatsou et al., 2024; Mulawarman et al., 2023; Öztan Ulusoy and Cihangül, 2021). The present study also revealed that technical constraints limiting visual interaction can complicate the counselor's ability to fully understand the client. In online environments, the absence of nonverbal communication has been reported as one of the most significant challenges (Amos et al., 2020; Zeren, 2017; Zeren et al., 2022). Eye contact, in particular, plays a key role in communicating attention and empathy to the client, thereby strengthening trust and reinforcing the therapeutic alliance (Knapp and Hall, 2010). According to psychological counselors, eye contact is especially important in online counseling, as it helps convey to the client that they are understood, fosters a sense of reassurance, and facilitates greater engagement in the therapeutic process. Based on these findings, it is recommended that the technical infrastructure of online counseling services be enhanced to better support visual communication and that counselors receive training to develop their nonverbal communication skills in digital settings. To ensure a holistic therapeutic process, both verbal and nonverbal communication elements must be restructured and optimized for the online context.

The findings reflected that participants interpreted clients' individual characteristics as factors shaping how they experienced the quality and flow of online counseling sessions. In particular, demographic variables such as age and socioeconomic status, as well as personality traits such as anxiety levels, are among the key factors determining the quality of interaction established in online settings. Zeren (2017) reported that participants made sense of gender compatibility between counselor and client as shaping the therapeutic alliance and perceived clients' individual characteristics as influencing how the counseling process unfolds, noting that individuals with high levels of anxiety were understood to experience greater challenges in expressing themselves in online settings. Voluntary disclosure of personal issues—one of the fundamental principles of online counseling (Ardi et al., 2017)—plays a decisive role in determining the quality of the alliance between counselor and client (Kiriakaki et al., 2022). Moreover, factors such as age and socioeconomic status can affect clients' participation in online psychological counseling services. In particular, individuals with lower socioeconomic status who face limited access to technology may experience significant barriers to benefiting from such services (Poyrazli and Can, 2020). In this context, it is crucial to adopt sensitive, flexible, and personalized approaches in online psychological counseling that are tailored to the individual characteristics of clients. Clients' demographic profiles, personality traits, and expressed concerns should be considered fundamental variables in planning and structuring online counseling processes.

The findings of this study indicate that the online psychological counseling process contributes not only to clients but also significantly to the professional development of counselors. Participants frequently emphasized the opportunity to work with diverse client profiles as an enriching factor for their professional growth, highlighting the potential of online platforms to provide diversity and broaden experience. Counselors' reports of gaining experience in interacting with individuals from various cultural backgrounds suggest that online counseling fosters cultural sensitivity and supports the development of a more multidimensional perspective. Similarly, (Tuna and Haskan Avci 2023) reported that online counseling enabled counselors to interact with clients from different cities, particularly during the pandemic period, which may have played a key role in enhancing their awareness of cultural diversity. Consistent with this finding, Özdemir and Barut (2020) stated that online psychological counseling allows communication with individuals from different geographical, cultural, and demographic contexts, positively influencing counselors' professional growth. (Yüksel Sahin 2021) further noted that during the COVID-19 pandemic, counselors providing online services enhanced their capacity to use technology and digital tools, gained experience with a variety of counseling settings, and ultimately supported their professional development. Another important professional contribution of online counseling is the improvement of time management skills. While online counseling offers temporal flexibility and independence for both counselors and clients, poor time management may lead to negative outcomes. For instance, Mulawarman et al. (2023) found that flexible scheduling occasionally disrupted communication. Therefore, counselors should develop time management skills, which play a critical role in the success of online counseling. In general, online psychological counseling practices appear to support counselors' professional development by enabling them to work with diverse client groups, increasing their cultural awareness, and strengthening their time management skills.

## 5 Conclusion

This study examined psychological counselors' experiences with online psychological counseling from a multidimensional perspective, providing comprehensive data regarding the advantages, limitations, and professional implications of this practice. According to the participants' views, the most frequently emphasized advantage of online counseling was the ease of access for clients and the flexibility of time and location. In contrast, the most prominent disadvantages were technical problems (particularly internet connection disruptions) and ethical concerns (such as confidentiality and the establishment of trust). These findings indicate that, while online counseling services have strong advantages in terms of accessibility, they must be carefully structured with respect to infrastructure and ethical standards. The process of establishing a therapeutic alliance in online settings varies depending on client characteristics and the nature of digital interactions. While the absence of face-to-face communication may create a sense of artificiality for some clients, the online environment can provide a safer space for expression for those who are shy or concerned about privacy. In this context, the quality of the therapeutic relationship in online counseling is directly related to the client's individual characteristics and perceptions of the counseling environment.

The client's self-disclosure behavior is similarly shaped by the perceived sense of safety and the quality of the established relationship. Ensuring a sense of security on digital platforms increases the customer's willingness to share their inner world, while the absence of this security can limit the functionality of the consulting process. Furthermore, the limited observability of body language and physical cues (such as eye contact, gestures, and facial expressions) in online settings makes it more difficult to accurately assess the client's emotional state and can affect therapeutic interaction. Clients' individual characteristics are among the key variables that determine the functioning of the online counseling process and the manner in which the therapeutic alliance is formed. This underscores the need for online counseling services to be delivered through individualized and flexible approaches.

Finally, the online counseling process also contributes to psychological counselors' professional development. Working with diverse client profiles, encountering cultural diversity, and improving time management skills through digital platforms are highlighted as factors that enhance counselors' professional competence and job satisfaction. Overall, online psychological counseling practices create both new opportunities and responsibilities for clients and counselors. In this context, strengthening technological infrastructure, adapting ethical principles to digital environments, and developing intervention strategies sensitive to client characteristics are essential for improving the quality of these services.

## 6 Recommendations

Based on the findings of this study, various recommendations have been developed to enhance the functionality, ethical standards, and professional contributions of online psychological counseling applications. First, it is crucial to create user-friendly and secure digital infrastructures to address the connection issues and usability difficulties frequently reported by participants. Moreover, core issues such as confidentiality, crisis management, and trust-building should be revisited in the online context, and digital ethical protocols should be developed along with guidelines and training materials for counselors. Additionally, providing specialized training for counselors to accurately interpret nonverbal communication elements—such as eye contact, facial expressions, and body language—in online settings will help strengthen the therapeutic alliance. Personalizing the counseling process by taking into account clients' individual characteristics such as age, socioeconomic status, anxiety level, and type of problem will significantly contribute to online counseling applications.

Another important point is the integration of online counseling into professional development. Working with diverse client profiles, exposure to cultural diversity, and the development of time management skills should be incorporated into counselor training programs as valuable competencies. Finally, to better understand the dynamics of online counseling, more research focusing on client satisfaction, the continuity of the therapeutic alliance, and potential ethical risks should be encouraged. Comparative studies examining face-to-face and online counseling formats will also contribute to the identification of best practices in the field. These recommendations offer a framework for strengthening both the quality of practice and the ethical and professional standards of online psychological counseling services and serve as a guide for practitioners, educators, and policymakers.

## 7 Limitations and future research

This study makes a significant contribution to the field by revealing psychological counselors' experiences regarding the online counseling process. Nevertheless, certain limitations should be taken into account. The research was conducted with data obtained from only 14 counselors working in Türkiye, which limits the generalizability of the findings to other cultural contexts or to larger counselor populations. Moreover, as the data are based on participants' self-reports, their accounts may reflect interpretations shaped by social desirability or professional norms. Another limitation of the study is its exclusive focus on counselors' perspectives. Given that the therapeutic relationship is inherently a bidirectional process, incorporating clients' perspectives would provide a more holistic understanding. Furthermore, the study was limited to specific themes such as therapeutic alliance, self-disclosure, and nonverbal communication, leaving out other important dimensions such as intervention effectiveness, long-term outcomes, and cultural adaptation.

It is important for future research to be designed in a way that addresses these limitations. Studies conducted with larger and more diverse samples across different cultural, institutional, and technological contexts will provide deeper insights into various dimensions of online counseling. Employing mixed-methods designs that combine qualitative findings with quantitative data may yield more robust and comprehensive results. Including clients' experiences, satisfaction levels, and perceptions of the therapeutic alliance will enable a more multidimensional understanding of the process. Investigating the impact of specific digital tools and platforms on the therapeutic process may also offer valuable practical implications. Furthermore, in-depth examinations of ethical decision-making processes and crisis management strategies in online settings could enhance both counselors' professional competencies and the reliability of online counseling services. Taken together, such future research efforts will present a more comprehensive and evidence-based understanding of the evolving nature of online psychological counseling, thereby making a significant contribution to both the literature and professional practice.

## Data Availability

The original contributions presented in the study are included in the article/supplementary material, further inquiries can be directed to the corresponding author.
